# Autoimmune cerebellar ataxia with anti-Homer3 antibodies associated with herpesvirus infection: a case report and literature review

**DOI:** 10.3389/fimmu.2025.1709326

**Published:** 2026-01-12

**Authors:** De-Jin Song, Ran Dong, Yi-Ming Liu, Tan Wang, Jun Ma

**Affiliations:** 1 Department of Neurology, Qilu Hospital of Shandong University, Jinan, Shandong, China; 2 Department of Geriatrics, Qilu Hospital of Shandong University, Jinan, Shandong, China

**Keywords:** ACA, autoimmune cerebellar ataxia, case report, herpesvirus infection, Homer-3, rituximab

## Abstract

Autoimmune cerebellar ataxia (ACA) is a cerebellar syndrome mediated by autoimmune mechanisms. ACA is particularly rare, and cases of ACA with anti-Homer-3 antibodies associated with herpesvirus infection are even rarer. In this study, we report a case of a 15-year-old girl who was admitted with a one-month history of progressive vertigo and unsteady gait. Metagenomic next-generation sequencing (mNGS) of her cerebrospinal fluid (CSF) revealed eight sequences of human herpesvirus 7 (HHV-7). Anti-Homer-3 antibodies were detected in both serum and CSF samples. Following a series of immunotherapy, the patient showed improvements in dizziness and gait stability. However, her symptoms recurred during the tapering of corticosteroids. The patient developed three episodes of generalized seizures. Concurrently, gait instability significantly worsened. Repeat first-line immunotherapies including corticosteroid and IVIG were not effective. Rituximab was initiated and symptoms were partial improved. In this study, we present the clinical symptoms of this patient with anti-Homer-3 antibody-associated ACA, conduct long-term follow-up, and review relevant literature. Our aim is to enhance the understanding of this rare disease by summarizing key clinical features, thus providing valuable insights into the diagnosis and treatment of ACA.

## Introduction

Autoimmune cerebellar ataxia (ACA) represents a significant cause of sporadic cerebellar ataxia (1, 2). The core clinical manifestations of ACA include gait ataxia, limb ataxia, dysarthria, dizziness, and nystagmus (3). It is classified into paraneoplastic ACA and non-paraneoplastic ACA, with the latter encompassing glutamate decarboxylase (GAD) antibody-associated cerebellar ataxia, gluten ataxia (GA), and primary autoimmune cerebellar ataxia (PACA) (4). The term PACA was used to describe patients with suspected immune-mediated cerebellar ataxia in which neither a trigger nor any pathogenic neuronal antibodies have been discovered. According to diagnostic criteria proposed by the International Task Force in 2020, PACA is defined by: a predominantly acute or subacute pure cerebellar syndrome; MRI at presentation usually normal or may show primarily cerebellar vermian atrophy; and at least two indicators of autoimmunity, such as inflammatory cerebrospinal fluid (CSF), a history of autoimmune disease, or the presence of relevant autoantibodies (5). Homer-3 antibody-associated encephalitis is a rare etiology of PACA, initially identified by Zuliani et al. in 2007 in a patient with subacute cerebellar ataxia (6). In this study, we reported a new case of Homer-3 antibody-associated encephalitis, a 15-year-old girl presenting clinical relapse of cerebellar ataxia that developed following an infection with human herpesvirus 7 (HHV-7), and in whom rituximab was initiated after first-line immunotherapies were not well effective.

## Materials and methods

We retrospectively analyzed the patient’s clinical data, including medical history, auxiliary examinations, treatment process and follow-up. The serum and cerebrospinal fluid (CSF) were tested using a commercial cell-based assay (CBA) (Euroimmun, Lübeck, Germany) for the autoimmune cerebellitis antibodies. Then, we reviewed and analyzed all ACA cases associated with Homer-3 antibodies reported so far, summarizing the clinical characteristics and prognosis of this disease.

## Case presentation

A 15-year-old girl presented with a one-month history of progressive vertigo and unsteady gait. At the onset, she experienced several days of recurrent low-grade fever, peaking at 37.4°C. she denied headache, tinnitus, auditory impairment or visual disturbances. Her past medical history and family history were unremarkable. Neurologic examination revealed slurred and irregular speech. Cranial nerve assessment showed bilateral gaze-evoked nystagmus with a downbeat component, particularly evident during leftward gaze (**Supplementary Video 1**). The patient demonstrated brisk deep tendon reflexes in all extremities. Examination of coordinated movements revealed bilateral dysmetria and dysdiadochokinesia. Gait assessment showed cerebellar ataxia, and she was unable to perform tandem walking.

**Figure 1:**
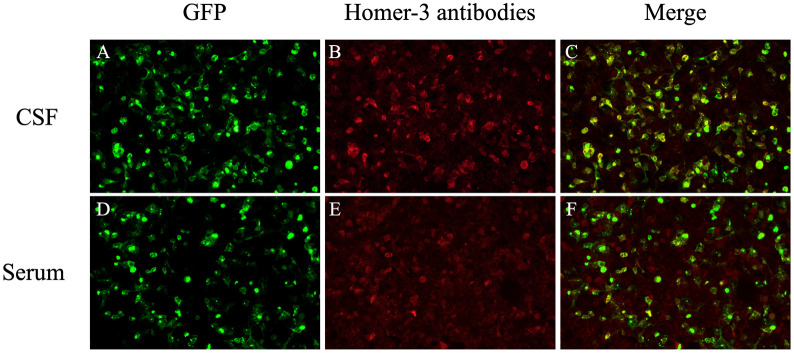
The Homer-3 antibodies of CSF and serum were positive (CBA, indirect immunofluorescence): **(A, D)** The green marker (GFP) represents Homer-3 antigen; The red marker represents anti-Homer-3 antibodies in CSF **(B)** and serum **(E)**; The image **(C)** and **(F)** show fluorescence overlap (positive colocalization).

**Figure 2:**
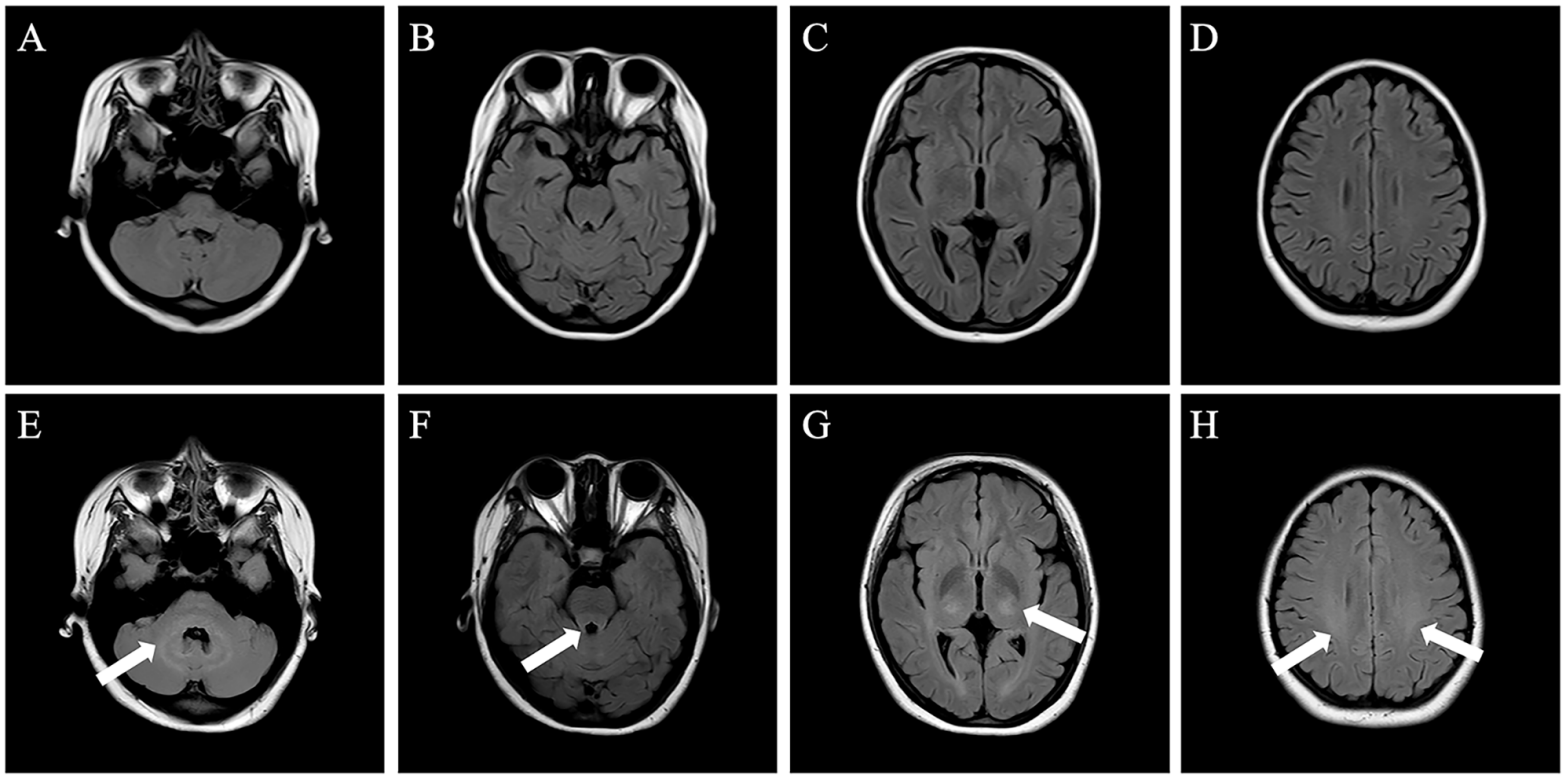
Initial and follow-up brain MRI (axial FLAIR sequence). **(A–D)** The initial brain MRI showed no structural abnormalities. **(E–H)** The follow-up brain MRI showed mild hyperintensities in bilateral cerebellar hemispheres **(E)**, dorsal aspect of the brainstem **(F)**, bilateral thalamic regions **(G)**, and along the corticospinal tract pathways **(H)**.

Laboratory investigations revealed elevated D-dimer (0.72 μg/mL). Complete blood count (CBC), biochemical tests, serum screening for systemic autoimmune diseases, tumor markers, vitamin B12 level and thyroid function were normal. Lumbar puncture demonstrated opening pressure of 130 mmH_2_O. CSF was lymphocytic pleocytosis (72 white blood cells/μL; 95% lymphocytes) with elevated protein (0.65 g/L; normal range 0.15–0.45g/L) and normal glucose and chloride levels. The oligoclonal band in the CSF was positive whereas the serum was negative. Metagenomic next-generation sequencing (mNGS) of CSF detected 8 unique reads of HHV-7. An autoimmune cerebellar ataxia panel, including anti-ATP1A3, ARHGAP26, AP3B2, CASPR2, GAD65, GluK2, Homer-3, IgLON5, ITPR1, KLHL11, mGluR1, mGluR2, PKCγ and PCA2, was conducted on both serum and CSF samples. Anti-Homer-3 antibody was positive in both serum (1:10) and CSF (1:1) (**Figure 1**). Autoimmune encephalitis-associated antibodies including NMDAR, LGI-1, CASPR2, GABAbR, AMPAR, and DPPX, as well as paraneoplastic antibodies including Hu, Yo, Ri, CV2, Ma2 and amphiphysin were also tested. The results were negative in both serum and CSF. Brain magnetic resonance imaging (MRI) revealed no structural abnormalities (**Figures 2A–D**). Screening for tumor was performed, including assessment of tumor markers, chest and abdomen CT scans, and gynecological ultrasound. No abnormalities were detected. Based on these findings, the patient was diagnosed with ACA with anti-Homer-3 antibodies. Treatment was started with intravenous immunoglobulin (IVIG, 25 g per day for 5 days) and intravenous methylprednisolone pulse therapy (500 mg daily for 5 days), followed by oral prednisone with a dose of 60 mg daily, gradually tapered by 5 mg every two weeks. Considering the temporal association with fever, the detection of HHV-7, the presence of CSF lymphocytic pleocytosis, and the exclusion of other identifiable causes of fever, HHV-7 infection was considered a plausible and likely explanation. Accordingly, antiviral treatment with intravenous acyclovir (0.5g every 8 hours) was administered for 14 days. The patient exhibited partial improvement in vertigo and unsteady gait.

### Follow-up

During the first follow-up visit, the patient continued to exhibit persistent gait instability. To prompt further immunological evaluation, lymphocyte subset analysis was performed, revealing a CD19^+^ B cell count of 483 cells/μL. To further improve the symptoms and reduce the risk of relapse, mycophenolate mofetil (MMF) was subsequently initiated at a dose of 0.5 g twice daily. Although mild improvement in gait was observed, an increase in CD19^+^ B cells count to 678 cells/μL at the second follow-up led to escalation of MMF to 0.75 g twice daily. Over the following month, the patient developed three episodes of generalized seizures without any obvious triggers, characterized by generalized limb convulsions, loss of consciousness, urinary incontinence, and tongue laceration. Consciousness was regained spontaneously within minutes after each episode. Concurrently, gait instability significantly worsened, resulting in the inability to ambulate independently. Therefore, the patient was readmitted to our hospital.

**Figure 3:**
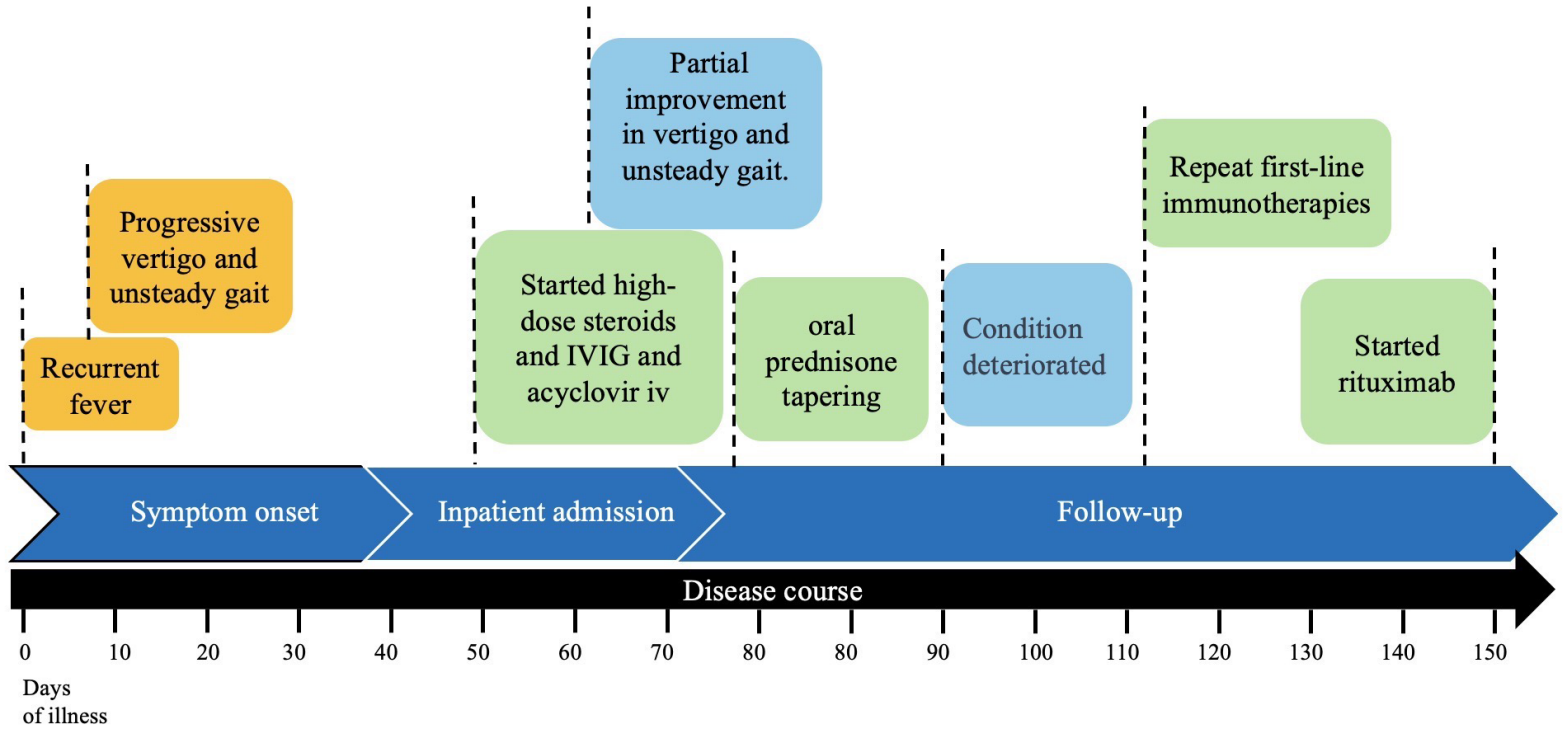
Timeline of clinical course and treatment regimens for case.

A repeat brain MRI revealed multiple abnormal signal intensities (**Figures 2E–H**). The main findings included T2 FLAIR hyperintensities in the bilateral cerebellar hemispheres, the dorsal aspect of the brainstem, bilateral thalamic regions, and along the corticospinal tract, without enhancement observed. Electroencephalography (EEG) showed mild abnormalities, including a reduction in the frequency of the bilateral posterior alpha rhythm to 8–9 Hz, with impaired amplitude modulation. Lumbar puncture demonstrated an opening pressure of 150 mmH_2_O. CSF analysis revealed lymphocytic pleocytosis (white blood cell count: 26 × 10^6^/L; 99% small lymphocytes) with elevated protein levels (0.71 g/L; normal range 0.15–0.45 g/L), and normal glucose and chloride levels. Repeat testing for both autoimmune cerebellar ataxia antibodies and demyelinating antibodies in serum and CSF yielded negative results. Additionally, vimentin-IgG testing was performed (at Xuanwu Hospital Capital Medical University), and the result was negative. The peripheral CD19^+^ B cell count was 460 cells/μL. The patient received IVIG and a second course of intravenous methylprednisolone pulse therapy using the same protocol as the initial treatment, along with MMF (0.75 g twice daily) and levetiracetam (500 mg twice daily). Following this intervention, seizure activity ceased, but gait instability showed minimal improvement. Given the suboptimal response to prior corticosteroid and IVIG therapy, rituximab was administered (100 mg on day 1, followed by 500 mg on day 2). After treatment, the patient’s gait instability improved, enabling independent ambulation. The timeline of the treatment is shown in **Figure 3**.

### Literature review

We reviewed and analyzed 16 reported cases of anti-Homer-3 antibody-associated ACA, summarizing the clinical presentations, CSF and imaging findings, treatment, and prognosis (**Table 1**) (6–17). The age at onset ranged widely from 10 to 84 years, with a median age of approximately 50 years and a female predominance (10/16, 62.5%). Prodromal symptoms were uncommon, observed in only two cases, which involved fever or cold. Cerebellar syndrome was the predominant clinical manifestation (15/16). Only one patient presented with non-cerebellar symptoms (Case 16: mild cognitive impairment and depression). Approximately 56.3% (9/16) of patients exhibited combined extra-cerebellar manifestations involving multiple systems, including: (1) neuropsychiatric symptoms: cognitive impairment (n = 3), psychiatric symptoms (n = 4), epileptic seizures (n = 2), and confusion (n=2); (2) rapid eye movement sleep behavior disorder (RBD) (n =2); (3) autonomic dysfunction: dysuria and orthostatic hypotension (n=2); and (4)spinal cord/peripheral nerve involvement (n = 2).

**Table 1:** Clinical features of 16 cases of anti-Homer-3 antibody positive autoimmune cerebellar ataxia.

Case No.	Gender/AAO	Onset form	Prodromal symptom	Clinical phenotype	Extra-cerebellar symptoms	Blood/CSF Homer3 antibody	CSF WBC (/ul)/protein (g/L)/intrathecal IgG synthesis	Tumor examination	Initial/follow-up MRI (months from onset)	Treatment	Follow-up
1 Zuliani L et al.,2007 (6)	F/65	Subacute	No	Cerebellar syndrome	–	(+)/NA	27/NA/CSF IgG index of 1.4 (normal < 0.7)	Negative	Normal/NA	Steroid	No improvement
2 HöftbergerR et al.,2013 (7)	M/38	Acute	No	Cerebellar syndrome and encephalopathy	Headache, confusion and seizures	(+)/NA	60/1.11/OB (–)	Negative	Normal/mild cerebellar atrophy (10)	IVIG and Steroid	Partially improved
3 Xu X et al.,2019 (8)	F/46	Insidious	No	Cerebellar syndrome	–	(+) 1:320/(+)	0/0.41/OB (+)	Negative	Cerebellar atrophy/cerebellar atrophy (48)	Steroid and MMF	Partially improved
4 Liu M et al.,2021 (9)	F/50	Subacute	No	Cerebellar syndrome, RBD and autonomic dysfunction	RBD; dysuria and postural hypotension	(+)/ (–)	2/0.30/OB (+)	Negative	Normal (2)/Cerebellum and pons atrophy with hot cross bun sign (16)	Steroid and MMF	Partially improved
5 Liu M et al., 2021 (9)	M/14	Subacute	Prodromal fever	Cerebellar syndrome, encephalopathy and myeloradiculopathy	Cognitive dysfunction; limb weakness and hyporeflexia, bilateral Babinski sign	(+), Anti-GM1 antibody weakly positive/ (–)	21/0.61/OB (+)	Negative	Diffuse T2 hyperintensity in bilateral cerebral hemispheres (1)/decrease of T2 hyperintensity (8)	Steroid and IVIG	Partially improved, relapsed twice during weaning from steroid
6 Liu M et al.,2021 (9)	M/65	Insidious	No	Cerebellar syndrome, RBD and autonomic dysfunction	RBD; dysuria and postural hypotension	(+)/ (–)	30/1.136/OB (–)	Negative	Cerebellum and pons atrophy (13)/cerebellum and pons and cerebellum peduncle atrophy with hot cross bun sign (24)	Steroid, IVIG, and plasma exchange	Condition deteriorated
7 Liu M et al.,2021 (9)	F/84	Subacute	No	Cerebellar syndrome	–	(+)/ (–)	6/0.48/NA	Potential malignant pulmonary nodules	Normal/normal (9)	Steroid	Condition stable
8 Liu M et al.,2021 (9)	F/59	Subacute	No	Cerebellar syndrome, encephalopathy, and radiculoneuropathy	Psychosis, seizure, confusion; limb weakness and hyporeflexia	(+)/ (–)	2/0.17/OB (–)	Negative	FLAIR and DWI hyperintensity in bilateral frontal and parietal cortex (9)/normal (10, after IVIG treatment)	Steroid and IVIG	Partially improved, relapse
9 Miao A et al.,2022 (10)	F/20	Acute	Cold	Cerebellar syndrome	Head shaking quickly from side to side	(+) 1:100/(+) 1:3.2	139/1.67/OB (+)	Negative	Right cerebellar hemisphere hyperintensity/normal (1.5)	Steroid, IVIG and MMF	Obviously improved
10 Xue J et al.,2022 (11)	F/49	Insidious	No	Cerebellar syndrome and RBD	RBD	(+) 1:32/NA	Normal/Normal/NA	Negative	Cerebellum and pons atrophy with hot cross bun sign/stable (8)	Steroid and MMF	Partially improved
11 Kuang Z et al.,2022 (12)	M/10	Acute	No	Cerebellar syndrome and encephalopathy	Cognitive impairment and irritability	(–), VGCC antibody (+)/(+) 1:1	30/0.3/NA	Negative	T2 and FLAIR hyperintensities in cerebellar hemispheres and vermis/mild cerebellar atrophy (12)	Steroid and IVIG	Obviously improved
12 Klötzsch C et al.,2022 (13)	F/58	Subacute	No	Cerebellar syndrome	–	(+) 1:32000/(+) 1:100	11/NA/CSF IgG index 0.89	Breast adenocarcinoma	Normal/NA	Steroid, IVIG and plasma exchange	Partially improved
13 Jia J et al.,2022 (14)	M/55	Subacute	No	Cerebellar syndrome	–	(+) 1:100/(+) 1:1	Normal/Normal/OB (–)	Negative	Normal/NA	Steroid and IVIG	Partially improved
14 Wu Q et al.,2022 (15)	M/25	Subacute	No	Cerebellar syndrome and psychiatric symptoms	Psychiatric symptoms	(+) 1:100/NA	50/0.63/increased CSF IgG index	Negative	FLAIR hyperintensities in cerebellar hemispheres and vermis/worsened cerebellar lesions with enhancement and obvious cerebellar atrophy (5)	Steroid, plasma exchange and rituximab	Steroid (deterioration), plasma exchange (partial improvement), relapse treated with rituximab (partial improvement)
15 Hu Y,et al.,2022 (16)	F/26	Subacute	No	Cerebellar syndrome	–	(+)/(+)	NA	Negative	Cerebellar swelling with enhancement/cerebellar atrophy (2)	Aggressive immunosuppression (undefined)	Limited improvement
16 Hansen N et al.,2023 (17)	F/54	Insidious	No	Mild cognitive impairment and depressive symptoms	Mild cognitive impairment and depressive symptoms	(+) 1:320/(+) 1:3.2	0/NA/NA; elevated phosphorylated tau protein 181	Negative	Cerebellar atrophy/NA	Repetitive transcranial magnetic stimulation treatment	No improvement

AAO, age at onset; F, female; M, male; FLAIR, fluid-attenuated inversion recovery; DWI, diffusion weighted imaging; IVIG, intravenous immunoglobulin; MMF, mycophenolate mofetil; NA, not available; OB, oligoclonal bands; RBD, rapid eye movement sleep behavior disorder; CSF, cerebrospinal fluid.

All patients were positive for anti-Homer-3 antibodies in serum and/or CSF. Two patients had co-existing autoantibodies, with anti-GM1 in Case 5 and VGCC antibody in Case 11. Cerebrospinal fluid analysis in the majority of patients showed pleocytosis, elevated protein levels, and positive oligoclonal bands, although normal CSF findings were also observed. Only two patients had potential tumor findings (Case 7: pulmonary nodules of suspected malignancy; Case 12: breast adenocarcinoma).

Initial brain MRI was performed in all patients and revealed variable findings, including normal MRI in 6 cases, cerebellar or pontine atrophy in 4 cases, cerebral hemispheric signal abnormalities in 2 cases, and cerebellar signal changes in 4 cases. Follow-up MRI was available in 12 patients at 1.5-48 months from onest. The abnormal singals improved in 3 patients following immunotherapy, whereas one patient showed worsened cerebellar lesions. Newly developed or worsen cerebellar atrophy was observed in 6 patients.

All patients except one received immunotherapy. Glucocorticoids combined with intravenous immunoglobulin (IVIG) was the first-line treatment, while some patients were treated with combined mycophenolate mofetil (MMF) (n = 4), plasma exchange (n = 3), or rituximab (n = 1). The prognosis for anti-Homer-3 antibody-associated ACA is relatively poor, with nearly all patients experiencing residual neurological deficits of varying severity.

## Discussion

ACA is a cerebellar dysfunction syndrome caused by autoimmune mechanisms and is classified into paraneoplastic ACA and non-paraneoplastic ACA (18). Paraneoplastic ACA is primarily caused by tumor-induced abnormal autoimmunity, leading to cross-reactivity that targets the cerebellum. The main associated antibodies include anti-Yo, anti-Hu, anti-Ri, anti-Tr, anti-CV2, anti-Ma2, and anti-voltage-gated calcium channels (VGCC) antibodies. Non-paraneoplastic ACA mainly includes GAD antibody-associated cerebellar ataxia, GA, and PACA (4). The etiology of PACA is generally unknown, or the pathogenic antibodies are unclear, which includes anti-Homer-3 antibodies (5).

Homer-3 antibody-associated encephalitis is a rare etiology of ACA. Homer-3 is highly expressed on Purkinje cell dendrites spines. It is the scaffold protein interacting with metabotropic glutamate receptor 1 (mGluR1) and intracellular calcium channel (ITPR1), thereby controlling the ability of the mGLuR1 receptor to trigger calcium responses (19). The presence of anti-Homer-3 antibodies in the human body specifically binds to Homer-3 proteins, disrupting the interaction between metabotropic and ionotropic glutamate receptors. This disruption impedes glutamate signal transduction, predominantly affecting cerebellar Purkinje cells and leading to cerebellar ataxia (20).

More and more studies have reported post-infectious autoimmune encephalitis is remarkably common after herpesvirus encephalitis, particularly herpes simplex virus infection (21, 22). In this patient, laboratory investigations revealed the presence of anti-Homer-3 antibody along with detection of HHV-7 DNA in CSF. HHV-7 is a widespread double-strand DNA virus in the human population that belongs to the β-herpes virinae subfamily and replicates in CD4^+^ T lymphocytes (23). There have been reports in the literature that HHV-7 infection is associated with autoimmune encephalitis with anti-NMDAR and anti-AMPAR antibodies (24), limbic encephalitis (25), and overlapping autoimmune syndromes (26). Interpreting these results is complex, as neurotropic viruses may cause anti-NMDAR encephalitis through several mechanisms. It is speculated that one such mechanism involves the lysis of neurons by neurotropic viruses, which releases antigens that stimulate antibodies against the NMDA receptor, thereby triggering autoimmune encephalitis. Another hypothesis suggests that anti-NMDAR encephalitis may stimulate the reactivation of latent viruses, leading to secondary encephalitis (24). HHV-7 primary infection typically occurs in early childhood, often presenting as roseola or remaining asymptomatic, and the virus establishes lifelong latency in lymphocytes thereafter. While reactivation is more common in immunocompromised individuals, emerging evidence indicates that HHV-7 can reactivate in immunocompetent hosts, occasionally causing febrile illness and neurological complications (27–30). In our 15-year-old immunocompetent patient, the acute onset fever preceding cerebellar ataxia, combined with CSF detection of HHV-7 DNA and exclusion of alternative etiologies, suggests that HHV-7 reactivation may have precipitated the development of autoimmune cerebellar ataxia. This proposed association aligns with mechanisms of virus induced autoimmunity, where viral exposure can trigger cross-reactive immune responses against neuronal antigens. However, we acknowledge that definitive causality cannot be established solely based on these findings, as HHV-7 detection could theoretically reflect a bystander effect (e.g., BBB disruption due to early autoimmune processes allowing latent virus entry into CSF). Confirmatory CSF PCR or serological assays (e.g., HHV-7 IgM/IgG) should be performed to confirm the diagnosis of active HHV-7 infection.

The presentation of epileptic seizures in this case represents a rare manifestation of Homer-3 autoimmune cerebellitis, with only two previously reported cases worldwide (7, 9).Our report constitutes the third documented instance. Antibody-mediated encephalitis disorders are frequently associated with seizures. Research indicates that approximately 27% of patients develop secondary autoimmune encephalitis, within two months following herpes simplex encephalitis, these cases commonly present with seizures and psychiatric or behavioral abnormalities (22). Brain MRI findings showed that 82% of these patients exhibit extensive areas of contrast enhancement, which is rare among cases with classical antibody-mediated encephalitis. These findings suggest that entry of complement and other proinflammatory molecules through a disrupted blood-brain barrier may contribute to epileptogenesis and worse outcome (22).

Oculomotor abnormalities are a hallmark of cerebellar dysfunction, among which downbeat nystagmus is a typical form characterized by slow upward eye movement and rapid downward saccade. This type of nystagmus is typically associated with dysfunction of the vestibulocerebellum, particularly the flocculus and paraflocculus (31). In our patient, we observed gaze-evoked nystagmus with a downbeat component. Such an ocular motor finding has rarely been reported as a typical manifestation of Homer-3 antibody-associated cerebellar ataxia. To date, only two cases with confirmed Homer-3 antibody positivity have been documented in the literature (12, 13).

There are no established standards for the treatment of Homer-3 antibody-associated encephalitis. Similar to other autoimmune encephalitis, first-line immunotherapies in the acute phase, including corticosteroids, IVIG, and plasma exchange, may be beneficial (15). In addition, long-term immunosuppression such as oral prednisone and MMF has been reported to halt or minimize cerebellar ataxia in some patients, providing long-term clinical benefit (8). In the patient we reported, treatment with corticosteroids and IVIG during the initial admission was effective; however, the patient relapsed during the tapering of corticosteroids, even while receiving conconmitant MMF. A second course of corticosteroids and IVIG failed to improve the symptoms. Given this lack of response, the patient was given intravenous rituximab and partial improvement was observed. This suggests that more intensive immunotherapy, such as rituximab, may be considered as a second-line option when the first-line treatment are ineffective (15).

The prognosis for anti-Homer-3 antibody-associated ACA is relatively poor, with nearly all patients experiencing residual impairments of varying severity. Among the 16 reported cases, only two achieved a favorable prognosis, while three showed no improvement, and one patient deteriorated. Furthermore, clinical relapses occurred in four cases, including the present one, following the tapering or discontinuation of corticosteroids, or the cessation of IVIG. Symptoms worsened after relapse, and disabilities persisted despite treatment, suggesting that recurrent disease courses may contribute to unfavorable outcomes.

The main limitation of the case is that HHV-7 DNA was detected in the patient’s CSF solely through mNGS, without confirmatory testing via CSF-specific PCR or serological assays such as HHV-7 IgM/IgG. Although our patient showed no clinical evidence of immunodeficiency, we acknowledge that more advanced immunological tests (e.g., detailed T/B cell functional assays or genetic testing for rare primary immunodeficiencies) were not performed. Given the rarity of HHV-7 encephalitis in immunocompetent individuals, future studies may benefit from comprehensive immunological profiling to further explore potential subtle immune abnormalities that may predispose to viral reactivation and CNS involvement. Additionally, considering the prominent cerebellar ataxia, bilateral pyramidal tract involvement on neuroimaging, and inadequate response to standard immunotherapies, vimentin-IgG associated meningoencephalomyelitis remains a differential diagnosis (32). Although the vimentin-IgG testing was negative, this potential diagnosis should still be included in future follow-up evaluations.

## Conclusions

We present a case of anti-Homer-3 antibody associated ACA in an immunocompetent adolescent that may have been precipitated by HHV-7 reactivation, supported by temporal correlation between fever and neurological symptoms, detection of replicative HHV-7 in CSF, exclusion of alternative etiologies, and biological plausibility. To our knowledge, this is the first documented case linking autoimmune cerebellar ataxia to HHV-7. Further studies are needed to explore the potential causal link between HHV-7 and autoimmune cerebellar ataxia. In cases of ACA recurrence, if the therapeutic response to combined glucocorticoids and IVIG is suboptimal, rituximab may be considered as a treatment option when clinically indicated.

## Data Availability

The original contributions presented in the study are included in the article/**Supplementary Material**. Further inquiries can be directed to the corresponding author/s.

## Supplementary material

Supplementary Video 1 The video showed bilateral gaze-evoked nystagmus.
